# Comprehensive Screening of Mouse T-Cell Epitopes in Human Herpesvirus 6B Glycoprotein H/L/Q1/Q2 Tetramer Complex

**DOI:** 10.1155/2020/4697529

**Published:** 2020-07-26

**Authors:** Mie Okutani, Akiko Kawabata, Mitsuhiro Nishimura, Satoshi Nagamata, Soichiro Kuwabara, Yasunari Haseda, Lisa Munakata, Ryo Suzuki, Yasuko Mori, Taiki Aoshi

**Affiliations:** ^1^BIKEN Center for Innovative Vaccine Research and Development, The Research Foundation for Microbial Diseases of Osaka University, 3-1 Yamadaoka, Suita, Osaka 565-0871, Japan; ^2^Vaccine Dynamics Project, BIKEN Innovative Vaccine Research Alliance Laboratories, Research Institute for Microbial Diseases, Osaka University, 3-1 Yamadaoka, Suita, Osaka 565-0871, Japan; ^3^Division of Clinical Virology, Center for Infectious Diseases, Kobe University Graduate School of Medicine, 7-5-2 Kusunoki-cho, Chuo-ku, Kobe, Hyogo 650-0017, Japan; ^4^Department of Obstetrics and Gynecology, Kobe University Graduate School of Medicine, 7-5-2 Kusunoki-cho, Chuo-ku, Kobe, Hyogo 650-0017, Japan; ^5^Laboratory of Drug and Gene Delivery Research, Faculty of Pharma-Science, Teikyo University, 2-11-1 Kaga, Itabashi-ku, Tokyo 173-8605, Japan; ^6^Department of Cellular Immunology, Research Institute for Microbial Diseases, Osaka University, 3-1 Yamadaoka, Suita, Osaka 565-0871, Japan

## Abstract

Human herpesvirus 6 (HHV-6) infects over 90% of people. The HHV-6 subtype, HHV-6B in particular, is often associated with exanthem subitum in early childhood. Exanthem subitum is usually self-limiting and good prognosis disease; however, some infants primarily infected with HHV-6B develop encephalitis/encephalopathy, and half of the patients developed encephalopathy reported to have neurological sequelae. Furthermore, after primary infection, HHV-6B remains in a latent state and sometimes reactivated in immunosuppressed patients, causing life-threatening severe encephalopathy. However, effective immunotherapies or vaccines for controlling HHV-6B infection and reactivation have not yet been established. Recently, we have found that the HHV-6B tetrameric glycoprotein (g) complex, gH/gL/gQ1/gQ2 is a promising vaccine candidate, and currently under preclinical development. To confirm our vaccine candidate protein complex induce detectable T-cell responses, in this study, we comprehensively screened CD4^+^ and CD8^+^ T-cell epitopes in the gH/gL/gQ1/gQ2 tetrameric complex protein in mice immunisation model. Both BALB/c and C57BL/6 mice were immunised with the tetrameric complex protein or plasmid DNA encoding gH, gL, gQ1, and gQ2, and then restimulated with 162 20-mer peptides covering the whole gH/gL/gQ1/gQ2 sequences; multiple CD4^+^ and CD8^+^ T-cell-stimulating peptides were identified in both BALB/c and C57BL/6 mice. Our study demonstrates that gH/gL/gQ1/gQ2 tetramer-targeted vaccination has potential to induce T-cell responses in two different strains of mice and supports the future development and application of T-cell-inducing vaccine and immunotherapies against HHV-6B.

## 1. Introduction

Human herpesvirus 6 (HHV-6) belongs to the *β*-herpesvirus subfamily and infects over 90% of people globally [1]. HHV-6 can be classified into two groups, variant A (HHV-6A) and variant B (HHV-6B), by their epidemiology and pathology [2, 3]. Although both HHV-6A and HHV-6B have been shown to be involved in human diseases [4, 5], HHV-6B infection is clearly associated with exanthem subitum in early childhood [1, 6]. Exanthem subitum is a self-limited disease with a good prognosis; however, it is relatively highly associated with febrile seizure. Although it is rare, some infants develop encephalitis/encephalopathy associated with HHV-6B infection. About half of children with encephalitis or encephalopathy are reported to have neurological sequelae in Japan [7]. Furthermore, after primary infection, HHV-6B remains in a latent state and is sometimes reactivated in immunosuppressed patients with severe encephalopathy [8–10]. However, there is no specific drug, no vaccines, and no cellular immunotherapy for controlling HHV-6B infection and reactivation has been established.

HHV-6B glycoprotein (g) H/gL/gQ1/gQ2 tetrameric complex has been shown to bind human CD134 (also called OX40) and be expressed on activated T lymphocytes [11]. gQ1 and gQ2 subunits were shown to be sufficient for CD134 binding, and a region in gQ1 was required for its function [12]. Regarding gH and gL subunits, a number of studies in other herpesviruses have shown that gH and gL subunits are involved in penetration and cell-to-cell spread [13, 14]. The gQ1 and gQ2 nucleotide sequences are shared only 70% between HHV-6A and HHV-6B, while the gH and gL genes are mostly conserved (approximately 90%) between these two variants; in fact, the gH and gL sequences are also conserved even among other herpesvirus families [15–18]. Monoclonal antibodies (MAbs) against HHV-6B gH and gQ1 were found to specifically neutralise virus infection [19, 20]. Recently, we have found HHV-6B gH/gL/gQ1/gQ2 tetrameric complex is one of the most attractive vaccine targets for controlling HHV-6B infection (Wang et al. manuscript submitted).

In a previous study, we identified that HHV-6B gQ1 protein-induced CD4^+^ and CD8^+^ T-cell responses by immunising BALB/c mice using DNA vaccination [21]. In this study, we more comprehensively screened T-cell responses against gH/gL/gQ1/gQ2 complex of HHV-6B by using a library of 162 peptides covering the whole gH/gL/gQ1/gQ2 protein sequences by using two strains of mice (BALB/c and C57BL/6) with two immunisation methods. These methods were intradermal protein injection of gH/gL/gQ1/gQ2 complex together with CpG adjuvant (protein vaccination), and intravenous injection of the corresponding plasmid DNA formulated with polyethylenimine (PEI) (DNA vaccination). The results revealed the successful identification of multiple CD4^+^ T-cell and CD8^+^ T-cell epitopes in both BALB/c and C57BL/6 mice, suggesting that vaccination targeting the gH/gL/gQ1/gQ2 complex can induce detectable T-cell responses irrespective of the strain of mice. Our results support the future application of gH/gL/gQ1/gQ2 complex for T-cell-inducing vaccines and immunotherapies against HHV-6B. The difference in T-cell responses between the two strains of mice and between the two vaccine systems is also discussed.

## 2. Results

### 2.1. Detection of CD4^+^ and CD8^+^ T-Cell Response Induction upon Immunisation with the Tetrameric Complex Protein

First, we performed immunisation with the gH/gL/gQ1/gQ2 tetrameric protein complex together with our developed CpG adjuvant named D35/DOTAP [22], which efficiently induces both MHC Class I and Class II immune responses against a variety of antigens including OVA, influenza HA split vaccine, and LLO91-99 peptide (Supplementary Figure 1). To identify CD4^+^ or CD8^+^ T-cell responses against gH/gL/gQ1/gQ2 tetrameric protein, gH/gL/gQ1/gQ2 tetrameric protein-immunised splenocytes from six mice were pooled and appropriately prepared (CD4^+^ or CD8^+^ T-cell-depleted, and undepleted whole splenocytes). Then, they were stimulated in vitro with each of 162 peptides covering all gH/gL/gQ1/gQ2 sequences and IFN-*γ* production was measured by ELISA. Due to both the variation of interexperimental IFN-*γ* production differences and some T-cell responses against cryptic T-cell epitopes [23] were not always consistently detected, we repeated this experiment three times. We also utilized *Z*-scoring normalization method [24] to marge and better visualize IFN-*γ* production data against 162 peptides from these three independent experiments. Of note, in our previous study, gQ1-expressing plasmid vaccination induced at least one CD4^+^ T-cell response and one CD8^+^ T-cell response in gQ1 protein in BALB/c mice [21], so we also expected that immunisation with gH/gL/gQ1/gQ2 tetrameric protein complex with CpG adjuvant would also induce at least one CD4^+^ T-cell response and one CD8^+^ T-cell response.

**Figure 1:**
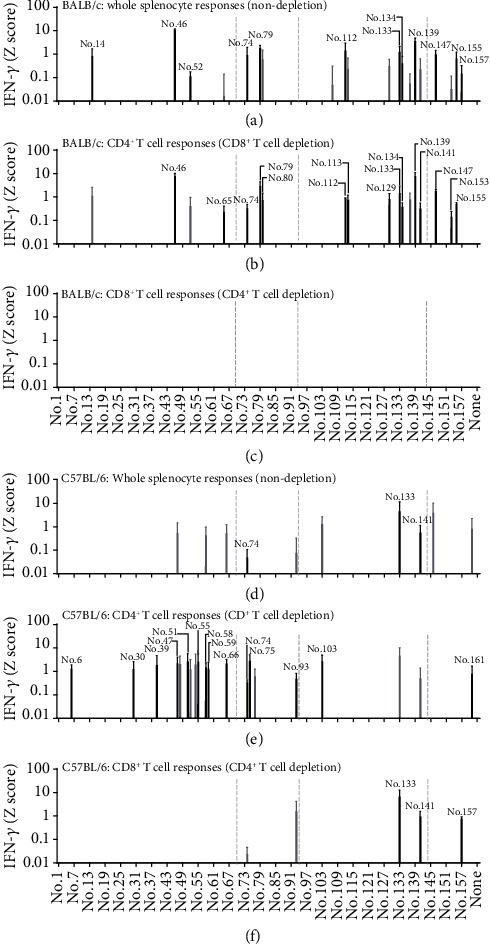
T-cell responses of mice immunised with the tetrameric protein complex plus CpG adjuvant. Undepleted whole splenocytes (a, d), CD8^+^ T-cell-depleted splenocytes (b, e), and CD4^+^ T-cell-depleted splenocytes (c, f) of BALB/c (a–c) and C57BL/6 (d–f) mice that had been immunised with HHV-6B gH/gL/gQ1/gQ2 tetrameric protein complex plus CpG adjuvant were tested for reactivity to a library of 162 peptides after 20 h of stimulation by IFN-g ELISA. The quantity of IFN-*γ* (pg/mL) of each peptide was transformed to a *Z* score (*Z* = x − mean/SD, where *x* is the quantity of IFN-*γ* for each peptide). Individual peptide samples with a *Z* score exceeding 0.01 were considered positive. Black bars indicate that the response was detected as positive in all three independent experiments, and gray bars indicate that there was positivity in two out of three independent experiments. T-cell responses detected as positive in only one experiment are not shown. Data are shown as the mean *Z* score ± SD of three independent experiments. The gray dot lines indicate the boundaries of gH, gL, gQ1, and gQ2.

Our results revealed IFN-*γ* production in whole splenocytes against a total of 12 peptides out of the library of 162 peptides covering the gH/gL/gQ1/gQ2 tetrameric proteins (see Supplementary Table 1) in BALB/c mice (Figure 1(a)). These 12 responses were consistently observed from our three independent experiments and indicated as black bars in Figure 1. Among them, a total of 9 peptides, namely, No. 46 (gH), No. 74 (gL), No. 79 (gL), No. 112 (gQ1), No. 133 (gQ1), No. 134 (gQ1), No. 139 (gQ1), No. 147 (gQ2), and No. 155 (gQ2) peptides, were confirmed to induce CD4^+^ T-cell responses because these responses still remained after CD8^+^ T-cell depletion (Figure 1(b)). Our previously identified CD4 T-cell epitope containing the 20-mer peptide AGLLMVNNIFTVQARYSKQN [21] was also included as No. 139 in this study's result. Other CD4^+^ T-cell responses against No. 65 (gH), No. 80 (gL), No. 113 (gQ1), No. 141 (gQ1), and No. 153 (gQ2) emerged only after CD8^+^ T-cell depletion, suggesting that they are relatively weak T-cell-stimulating peptides or that a relatively small population of CD4^+^ T cells responds to these peptides (Figure 1(b)), because after CD8^+^ T-cell depletion, we used the CD8^+^ cell-depleted cells containing the same cell number of nondepleted cells for stimulation meaning that relative CD4^+^ T-cell frequency was increased after CD8^+^ cell depletion. Upon including peptides detected in two out of three independent experiments (indicated as gray bars in Figure 1), the overall responses of whole splenocytes (Figure 1(a)) and of CD4^+^ T cells (Figure 1(b)) generally overlapped. In contrast and unexpectedly, no consistent CD8^+^ T-cell responses were detected (Figure 1(c)). Notably, responses against No. 157 (gQ2) were only observed with whole splenocytes but disappeared after CD4^+^ or CD8^+^ T-cell depletion.

In C57BL/6 mice, consistent (meaning three times out of three independent experiments) IFN-*γ* production was only seen against No. 74 (gL), No. 133 (gQ1), and No. 141 (gQ1) (Figure 1(d)). After CD8^+^ T-cell depletion, many weak but consistently detectable CD4^+^ T-cell responses were raised (Figure 1(e)). Unlike in BALB/c mice, CD8^+^ T-cell responses were consistently detected against No. 133 (gQ1), No. 141 (gQ1), and No. 157 (gQ2) by the protein vaccination in C57BL/6 mice, suggesting that protein immunisation with CpG adjuvant potentiated CD8^+^ T-cell responses as expected, at least in C57BL/6 mice.

### 2.2. T-Cell Response Induction upon Immunisation with DNA Vaccine

**Figure 2:**
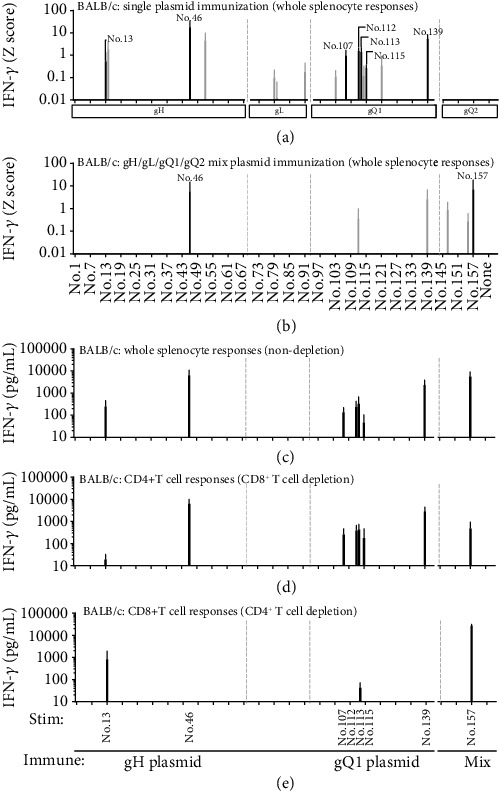
T-cell responses of immunised BALB/c mice upon vaccination with expression plasmid DNA. Undepleted whole splenocytes immunised with each expression plasmid for gH, gL, gQ1, and gQ2 (a), or a mixture of them (b) were tested for the corresponding library peptides (gH: No. 1–69, gL: No. 70–93, gQ1: No. 94–144, and gQ2: No. 145–162). After 20 h of stimulation, the quantity of IFN-*γ* (pg/mL) for each peptide was measured by ELISA and transformed to a *Z* score (*Z* = x − mean/SD, where *x* is the quantity of IFN-*γ* for each peptide). Individual peptide samples with a *Z* score exceeding 0.01 were considered as positive. Black bar indicates three detections, and gray bar indicates two detections out of three independent experiments. Data are shown as the mean *Z* score ± SD of three independent experiments. Consistently detected peptides shown by black bars in (a) and (b) were chosen and another independent experiment was performed. Undepleted whole splenocytes (c), CD8^+^ T-cell-depleted splenocytes (d), and CD4^+^ T-cell-depleted splenocytes (e) were tested for reactivity to the chosen peptides. After 20 h of stimulation, the production of IFN-*γ* was measured by ELISA. Data are presented in pg/mL and shown as mean ± SD. The gray dot lines indicate the boundaries of gH, gL, gQ1, and gQ2.

Because protein vaccination with CpG adjuvant in BALB/c mice unexpectedly induced no detectable CD8^+^ T-cell responses (Figure 1(c)), we also attempted plasmid DNA vaccination to more comprehensively screen T-cell responses against the gH/gL/gQ1/gQ2 tetrameric complex antigen. Mice were immunised with plasmids expressing each of gH, gL, gQ1, and gQ2, or a gH/gL/gQ1/gQ2 plasmid mixture, and then whole splenocytes from 2 mice per group were pooled and stimulated with the corresponding library peptides for each immunised plasmid (gH: No. 1–69, gL: No. 70–93, gQ1: No. 94–144, and gQ2: No. 145–162); subsequently, IFN-*γ* production was assayed by ELISA. This experiment was also performed three times independently, and the results were visualized similarly as Figure 1. IFN-*γ* production was consistently detected against No. 13, No. 46 (gH), No. 107, No. 112, No. 113, No. 115, and No. 139 (gQ1) peptides when BALB/c mice were immunised with single gH or gQ1 plasmid (Figure 2(a)). Interestingly, immunisation with gH/gL/gQ1/gQ2 plasmid mixture induced consistent T-cell responses only against two peptides, namely, No. 46 (gH) and No. 157 (gQ2) (Figure 2(b)), but not against other peptides, which induced such responses upon single plasmid immunisation, such as No. 13, No. 107, No. 112, No. 113, No. 115, and No. 139 (Figure 2(a)). T-cell responses against No. 157 peptide were only detected upon immunisation with gH/gL/gQ1/gQ2 plasmid mixture (Figure 2(b)). Immunisation with the single gQ2 plasmid did not induce any detectable T-cell responses against No. 157 peptide throughout three independent experiments (Figure 2(a)).

**Figure 3:**
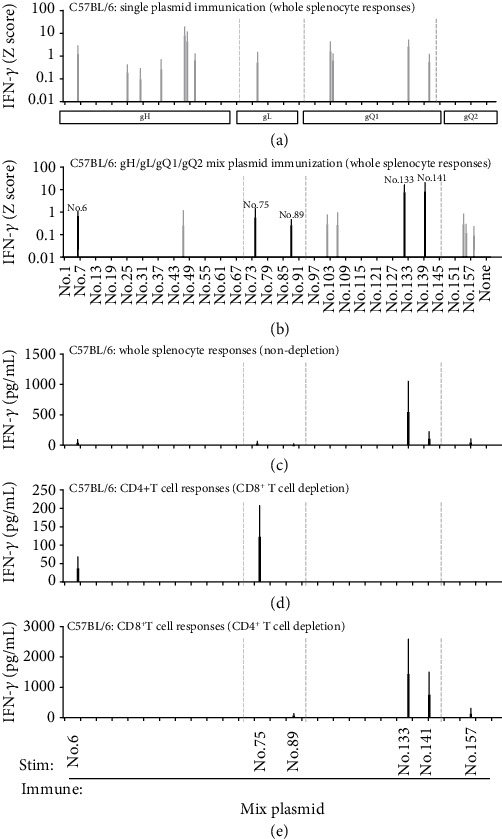
T-cell responses of C57BL/6 mice upon immunisation with expression plasmid DNA. Undepleted whole splenocytes immunised with each expression plasmid for gH, gL, gQ1, and gQ2 (a), and a mixture of them (b) were tested for the corresponding library peptides (gH: No. 1–69, gL: No. 70–93, gQ1: No. 94–144, and gQ2: No. 145–162). After 20 h of stimulation, the quantity of IFN-*γ* (pg/mL) of each peptide was measured by ELISA and transformed into a *Z* score (*Z* = x − mean/SD, where *x* is the quantity of IFN-*γ* for each peptide). Individual peptide samples with a *Z* score exceeding 0.01 were considered positive. Black bar indicates three, and gray bar indicates two detections out of three independent experiments. Data are shown as mean *Z* score ± SD of three independent experiments. Consistently detected peptides as shown by black bars in (a) and (b) were chosen and another independent experiment was performed. Undepleted whole splenocytes (c), CD8^+^ T-cell-depleted splenocytes (d), and CD4^+^ T-cell-depleted splenocytes (e) were tested for reactivity to the chosen peptides. After 20 h of stimulation, the production of IFN-*γ* was measured by ELISA. Data are presented in pg/mL and shown as mean ± SD. The gray dot lines indicate the boundaries of gH, gL, gQ1, and gQ2.

In C57BL/6 mice, immunisation with each single plasmid gH, gL, gQ1, or gQ2 induced no consistent T-cell responses against the peptide library (Figure 3(a)); the gray bars indicate only two times detection from three independent experiments. On the other hand, immunisation with the plasmid mixture induced weak but consistent T-cell responses against No. 6 (gH), No. 75, No. 89 (gL), No. 133, and No. 141 (gQ1) peptides (Figure 3(b)). Notably, in both BALB/c and C57BL/6 mice, no T-cell responses were seen upon immunisation with gQ2 single plasmid, but detectable responses such as against No. 157 peptide in gQ2 were seen after immunisation with the plasmid mixture, suggesting that the gQ2-encoding plasmid was successfully expressed and worked as a DNA vaccine in vivo.

### 2.3. CD4^+^ and CD8^+^ T-Cell Response Induction upon Immunisation with DNA Vaccine

To determine whether the DNA vaccination induced responses were from CD4^+^ or CD8^+^ T cells, the splenocytes after DNA vaccination were CD4^+^ or CD8^+^ T-cell-depleted and then stimulated with the selected peptides that had induced consistent T-cell responses upon single or mixed plasmid immunisation as shown in Figures 2(a) and 2(b). In BALB/c mice, IFN-*γ* responses were detected against all eight examined peptides shown by the black bars in Figures 2(a) and 2(b), with particularly strong IFN-*γ* production for No. 46, No. 139, and No. 157 (Figure 2(c)). After CD8^+^ T-cell depletion, CD4^+^ T-cell responses were detected for No. 46, No. 107, No. 112, No. 113, No. 115, No. 139, and No. 157 peptides (Figure 2(d)). After CD4^+^ T-cell depletion, CD8^+^ T-cell responses were detected for No. 13, No. 113, and No. 157 (Figure 2(e)). Notably, No. 113 peptide contains AFCPMTSKL, which we previously identified as a 9-mer H2K^d^-restricted CD8^+^ T-cell epitope in BALB/c mice [21]; this demonstrated that gQ1 plasmid immunisation reproducibly induced similar CD8^+^ T-cell responses in BALB/c mice.

In C57BL/6 mice, similarly, whole splenocytes immunised with a mixture of four plasmids responded to No. 133 and No. 141 among the five examined peptides shown by the black bar in Figure 3(b) (Figure 3(c)). CD4^+^ T-cell responses were detected against No. 6 (gH) and No. 75 (gL) peptides after CD8^+^ T-cell depletion (Figure 3(d)). CD8^+^ T-cell responses were detected against No. 89, No. 133, No. 141, and No. 157 after CD4^+^ T-cell depletion; although, No. 89 and No. 157 were barely detectable (Figure 3(e)). From these results, we selected No. 13 and No. 157 peptides as new CD8^+^ T-cell-responsive peptides for BALB/c mice (Figure 2(e)) and No. 133, No. 141, and No. 157 peptides for C57BL/6 mice (Figure 3(e)), for more detailed CD8^+^ T-cell epitope examination.

### 2.4. Determination of CD8^+^ T-Cell Short Epitope Peptides in BALB/c Mice

**Figure 4:**
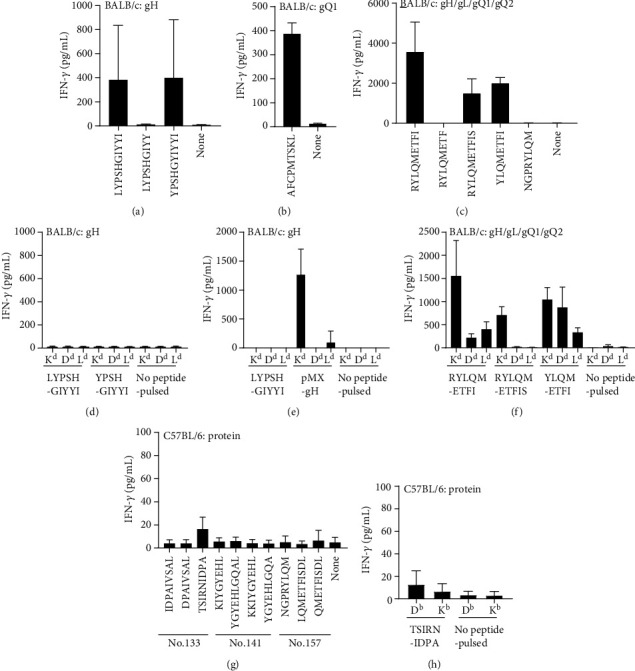
Short epitope determination for CD8^+^ T-cell-responding 20-mer peptides. Undepleted splenocytes of BALB/c mice immunised with expression plasmid of gH (a, d, e), gQ1 (b), and a mixture of gH, gL, gQ1, and gQ2 (c, f), and C57BL/6 mice immunised with the tetrameric protein complex plus CpG adjuvant (g, h) were tested with short peptides shown in Table 1 (a–c) and Table 2 (g). The restricted MHC I molecules were determined by the peptide-pulsed BW5147 cell lines expressing each of the H2d molecules (d–f) or H2b molecules (h). For LYPSHGIYYI restricted MHC I molecule determination, gH-expressing BW5147 cell lines were also examined (e). The production of IFN-*γ* after 20 h of stimulation was measured by ELISA. Data are presented in pg/mL and shown as mean ± SD.

To determine short CD8^+^ T-cell epitopes, several CD8^+^ T-cell short epitope candidates within the two peptides No. 13 and No. 157 were predicted using two computer programmes: BIMAS HLA Peptide Binding Prediction (https://www-bimas.cit.nih.gov/molbio/hla_bind/) and NetMHC 4.0 (http://www.cbs.dtu.dk/services/NetMHC/) (Table 1). Three short peptides within No. 13 and five short peptides within No. 157 that exhibited a high score or low % rank were synthesised (shown by the underline in Table 1). Undepleted whole splenocytes of BALB/c mice immunised with gH plasmid, gQ1 plasmid, or gH/gL/gQ1/gQ2 plasmid mixture were stimulated with these peptides. LYPSHGIYYI or YPSHGIYYI within No. 13 (gH) (Figure 4(a)) and RYLQMETFI within No. 157 (gQ2) (Figure 4(c)) provoked strong IFN-*γ* production among these candidate peptides, indicating that LYPSHGIYYI/YPSHGIYYI in gH and RYLQMETFI in gQ2 are the CD8^+^ T-cell short epitopes in BALB/c mice. For No. 133 peptide, we had already performed similar experiments and reported that No. 113 (gQ1) contained AFCPMTSKL, an H2K^d^-restricted CD8^+^ T-cell short epitope [21]. We confirmed that gQ1 plasmid immunisation with the PEI method also induced AFCPMTSKL-reactive T cells (Figure 4(b)).

To determine the restricted MHC I molecule of these responses, single MHC I-expressing cells such as BW5147-H2K^d^, -H2D^d^, and -H2L^d^ cells were pulsed with the short epitope peptide LYPSHGIYYI or YPSHGIYYI within No. 13 (gH) and RYLQMETFI within No. 157 (gQ2); then, they were used for the stimulation. Unexpectedly, all sets of BW5147-H2d-expressing cells pulsed with either LYPSHGIYYI or YPSHGIYYI within No. 13 (gH) resulted in no IFN-*γ* responses (Figure 4(d)). On the other hand, RYLQMETFI within No. 157 (gQ2) induced the strongest IFN-*γ* production in an H2K^d^-restricted manner (Figure 4(f)). To further investigate the failure of the short peptide-pulsed approach with No. 13 (gH) peptide (Figure 4(d)), we established internally gH-expressing BW5147-H2K^d^, -H2D^d^, and -H2L^d^ cells, and then used them for stimulation. In this approach, we successfully detected IFN-*γ* production by BW5147-H2K^d^-gH cells (Figure 4(e)). Taken together, these results suggested that LYPSHGIYYI or YPSHGIYYI within No. 13 (gH) was restricted by H2K^d^, and RYLQMETFI within No. 157 (gQ2) was also restricted by H2K^d^.

### 2.5. Determination of CD8^+^ T-Cell Short Epitope Peptides in C57BL/6 Mice

Since No. 133, No. 141 (gQ1), and No. 157 (gQ2) were found to induce CD8^+^ T-cell responses in C57BL/6 mice after both protein (Figure 1(f)) and plasmid immunisation (Figure 2(e)), several CD8^+^ T-cell short epitope candidates within these peptides were also predicted in the same way as mentioned above (Table 2). Three short peptides from No. 133, four short peptides from No. 141, and five short peptides from No. 157 associated with a high score or a low % rank were synthesised (shown by the underline in Table 2). Undepleted splenocytes from C57BL/6 mice immunised with protein were stimulated with these peptides. TSIRNIDPA within No. 133 (gQ1) induced IFN-*γ* production above the background level (Figure 4(g)), while no other epitope candidate peptides within No. 141 (gQ1) and No. 157 (gQ2) induced any discriminating responses (Figure 4(g)). In the MHC class I restriction determination, BW5147-H2D^b^ pulsed with TSIRNIDPA induced slightly stronger IFN-*γ* production compared with BW5147-H2K^b^ pulsed with TSIRNIDPA (Figure 4(h)). We also performed similar experiments using C57BL/6 mouse splenocytes immunised with a mixture of plasmids (Supplementary Figure 2A); however, even with CD4^+^ T-cell depletion, the results for both short peptides were not convincingly clear (Supplementary Figure 2B). Our results obtained in C57BL/6 mice did not provide a definitive answer regarding the restricted MHC I molecule of TSIRNIDPA. The responses to other short peptides in No. 141 (gQ1) and No. 157 (gQ2) were not confirmed. Taking the obtained findings together, we concluded that at least TSIRNIDPA in gQ1 is a CD8^+^ T-cell short epitope in C57BL/6 mice.

### 2.6. CD8^+^ T-Cell Response Induction by 20-Mer Peptide Vaccination

**Figure 5:**
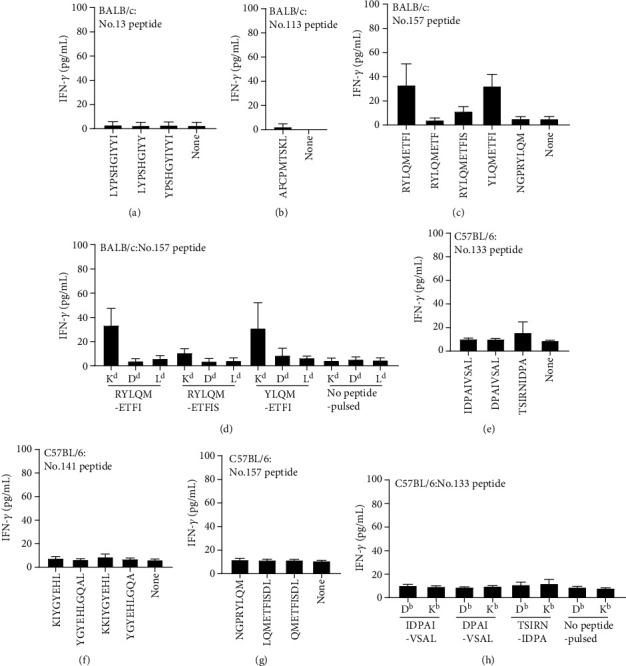
T-cell response inductions with 20-mer peptide plus CpG adjuvant. Whole splenocytes of BALB/c mice immunised with No. 13 (a), No. 113 (b), or No. 157 (c) peptide plus CpG adjuvant, and whole splenocytes of C57BL/6 mice immunised with No. 133 (e), No. 141 (f), or No. 157 (g) peptide plus CpG adjuvant were tested for the reactivity to CD8^+^ T-cell short epitope candidates. After 20 h of stimulation, the production of IFN-*γ* was measured by ELISA. Data are presented in pg/mL and shown as mean ± SD.

In gH/gL/gQ1/gQ2 tetrameric complex, thus far, we have identified three confirmed H2K^d^-restricted CD8^+^ T-cell epitopes in BALB/c mice and three potential CD8^+^ T-cell-stimulating 20-mer peptides in C57BL/6 mice. To determine whether these CD8^+^ T-cell epitope containing 20-mer peptides with CpG adjuvant (instead of whole protein or plasmid vaccination) could induce detectable CD8^+^ T-cell responses, we immunised BALB/c mice with No. 13, No. 113, or No. 157 peptides (20-mers), which included LYPSHGIYYI, AFCPMTSKL, and RYLQMETFI CD8^+^ T-cell epitopes, and C57BL/6 mice with No. 133, No. 141, or No. 157. In BALB/c mice, No. 157 peptide vaccination induced a detectable CD8^+^ T-cell response against RYLQMETFI (Figure 5(c)), while No. 13 and No. 113 peptide vaccination did not (Figures 5(a) and 5(b)). In C57BL/6 mice, No. 133 peptide vaccination induced a very weak CD8^+^ T-cell response against TSIRNIDPA (Figure 5(e)), but No. 141 and No. 157 peptide vaccination did not induce any detectable CD8^+^ T-cell responses (Figures 5(f) and 5(g)). Peptide vaccination induced RYLQMETFI/H2K^d^ responses in BALB/c mice (Figure 5(d)), and No. 133 peptide vaccination similarly induced CD8^+^ T-cell responses against TSIRNIDPA peptide (Figure 5(e)). However, again, the restricted MHC I molecules were still not clarified for TSIRNIDPA peptide (Figure 5(h)), potentially due to the weak binding to the corresponding MHC molecule and the resultant weak immune induction against this peptide in C57BL/6 mice.

## 3. Discussion

In this study, we identified multiple CD8^+^ and CD4^+^ T-cell-responding epitopes or 20-mer peptides in the HHV-6B gH/gL/gQ1/gQ2 tetrameric complex by using two different immunisation methods including protein and DNA vaccinations. In BALB/c mice, we identified that LYPSHGIYYI within No. 13 (gH) and RYLQMETFI within No. 157 (gQ2) are new H2K^d^-restricted CD8^+^ T-cell epitopes, and No. 46 (gH), No. 79 (gL), and No. 147 (gQ2) are relatively strong CD4^+^ T-cell-stimulating 20-mer peptides. Interestingly, LYPSHGIYYI and YPSHGIYYI within No. 13 (gH) were not stimulatory when they were pulsed on single H2K^d^-, H2D^d^-, or H2L^d^-expressing BW5147 cells (Figure 4(d)). On the other hand, gH protein-expressing BW5147-H2K^d^ cells (transduced with gH-expressing retrovirus vector) were strongly stimulatory (Figure 4(e)), suggesting that this T-cell epitope is more stably presented on H2K^d^ through the endogenous antigen processing pathway. By searching the mouse genome, mouse plexin-A3 contains LYPAFDIYYI sequence, which only differs in the middle part of the LYPSHGIYYI epitope, from SHG to AFD. This may contribute to the observed phenomenon, but further experiments are required to understand this.

In a previous study, we examined T-cell epitopes within gQ1 in BALB/c mice by DNA vaccination and found that AFCPMTSKL within P17 (No. 113 peptide in the library of this study) was the H2K^d^-restricted CD8^+^ T-cell epitope, and P43 (No. 139 peptide in this study) stimulated CD4^+^ T-cell responses. This study also confirmed these findings of a CD8^+^ T-cell response against No. 113 peptide and a CD4^+^ T-cell response against No. 139 peptide (Figures 1 and 2).

In C57BL/6 mice, we found that TSIRNIDPA within No. 133 (gQ1) is the CD8^+^ T-cell epitope, but we could not clearly determine the restricted H2b molecule for TSIRNIDPA short peptide. For No. 141 and No. 157, we consistently observed CD8^+^ T-cell responses against these 20-mer peptides, but these responses were somehow too weak to further determine the short peptide epitopes for them. No. 6, No. 39, No. 47, No. 51, No. 55, No. 66 (gH), No. 75 (gL), and No. 103 (gQ1) were CD4^+^ T-cell-stimulating 20-mer peptides in C57BL/6 mice.

By using two strains of mice (BALB/c and C57BL/6) and immunising them with two different methods (using protein and DNA), we also observed different T-cell responses between BALB/c and C57BL/6 mice, and between protein vaccine and DNA vaccine. Overall, T-cell responses were stronger in BALB/c mice than in C57BL/6 mice, irrespective of the immunisation method (Figures 1–3).

BALB/c and C57BL/6 mice have different MHC haplotypes. MHC haplotype of BALB/c is H-2d and that of C57BL/6 is H-2b. Different MHC haplotype molecules present different peptides to the T cells, so that T cells from BALB/c or C57BL/6 respond to different peptides is expected result from the view of MHC haplotype. Another possible factor that contributes the difference of T-cell responses between BALB/c and C57BL/6 mice is their preferences to develop Th1 or Th2 type cytokine response, respectively. They are regarded as prototypes of Th1 or Th2 mouse strains [25]. Recent papers also showed BALB/c mice have a tendency of Th2 type immune responses, and C57BL/6 mice Th1 type immune responses, especially in innate and immunometablic phases [26, 27]. These innate responses usually affect the adaptive immune responses, too. However, there is a report that BALB/c and C57BL/6 mice do not show default Th1 and Th2 preference in adaptive immune response by allergen immunisation [28]. The observed T-cell response difference between BALB/c and C57BL/6 in this study is mainly a result of MHC haplotype differences; however, it could be also influenced by Th1/Th2-related genetic background differences of these two strains.

In terms of the difference of vaccination methods, we observed a general tendency that protein vaccination preferentially induced CD4^+^ T-cell responses even with CpG adjuvants, and DNA vaccination preferentially induced CD8^+^ T-cell responses. This fits with the classical view of antigen presentation pathway that internal antigen preferentially presented on MHC-I for inducing CD8+ T-cell responses, and that external antigen preferentially presented on MHC-II for CD4+ T-cell responses. In other words, the DNA vaccination can provide internal antigen, and the protein vaccination largely provides external antigen. However, many reports also demonstrated that alternative “cross-presentation pathway” is a physiologically important pathway to induce CD8+ T-cell responses to infectious disease and cancer, in which external antigen is presented on MHC-I for inducing CD8+ T-cell responses [29, 30]. It is also known that using an adjuvant such as CpG in protein vaccination, the immunised protein antigen (this is external antigen) was also efficiently processed and induced CD8+ T-cell responses via this cross-presentation pathway.

We initially expected the induction of CD8^+^ T-cell response with protein + CpG adjuvant immunisation, because it has been shown that CpG adjuvant can induce strong CD8^+^T-cell responses for many protein vaccines, and we also observed the induction of CD8^+^ T-cell responses with CpG adjuvant for a variety of antigens (Suppl. Figure 1). This expectation was true for C57BL/6 mice. gH/gL/gQ1/gQ2 tetrameric protein + CpG adjuvant immunisation induced detectable CD8^+^ T-cell responses against No. 133, No. 141, and No. 157 peptides (Figure 1(f)). In C57BL/6 mice, CD8^+^ T-cell responses to the same three peptides were also similarly induced by the DNA vaccination method (Figure 3(e)). In contrast, this expectation was not met in BALB/c mice. Specifically, protein vaccination did not induce any consistently detectable CD8^+^ T-cell responses in these mice (Figure 1(c)). Instead, DNA vaccination induced consistently detectable CD8^+^ T-cell responses against No. 13, No. 113, and No. 157 peptides (Figure 2(e)). Interestingly, T-cell responses against No. 13 were only detected by single gH DNA immunisation in BALB/c mice. In contrast, T-cell responses against No. 157 in BALB/c mice were only induced by immunisation with a mixture of DNA (Figure 2(b)), but not with single gQ2 DNA (Figure 2(a)). In C57BL/6 mice, only immunisation with a mixture of DNA led to consistently detectable T-cell responses (Figure 3(b)). This may reflect the status differences of gH/gL/gQ1/gQ2 complex protein structures between single and mixed expression, and the antigen processing and presentation may be influenced by these protein structure-dependent effects. Although these issues need to be examined in future experiments, consistent CD8^+^ T-cell responses were only induced by DNA vaccination in BALB/c mice (Figure 2(e)). Similar phenomena have been reported for a tuberculosis vaccine model in mice. In this tuberculosis model, immunisation with rTB10.4 antigen + CAF05 adjuvant (potentially CD8^+^ T-cell response-inducing adjuvant composed of DDA/TDB/poly I: C) resulted in only CD4^+^ T-cell responses rather than the expected CD8^+^ T-cell responses, while the same antigen induced strong CD8^+^ T-cell responses with *M. tuberculosis* infection [31], suggesting that recombinant protein vaccination even with a potentially cross-presentation-activating adjuvant like CAF05 or CpG cannot always successfully induce the expected CD8^+^ T-cell responses. On the other hand, in a similar tuberculosis model using a different antigen, immunisation with a recombinant adenovirus vector induced mostly CD8^+^ T-cell responses and only weak CD4^+^ T-cell responses [32]. This situation is similar to the protein vs. DNA immunisation in this study. Importantly, in the tuberculosis model, CD4^+^ T-cell but not CD8^+^ T-cell responses were protective against challenge with *M. tuberculosis* infection [31, 32].

Although we could not examine the protective efficacy of the newly identified T-cell epitopes due to the lack of an established animal model for HHV-6B infection, our results demonstrated that an HHV-6B gH/gL/gQ1/gQ2 tetramer complex-targeted vaccine approach can induce multiple CD4^+^ and CD8^+^ T-cell responses irrespective of the immunised mouse strain, supporting that HHV-6B gH/gL/gQ1/gQ2 tetramer complex is a promising candidate to develop an HHV-6B vaccine.

## 4. Methods

### 4.1. Preparation of gH/gL/gQ1/gQ2 Protein Complex

The preparation of the gH/gL/gQ1/gQ2 tetrameric complex was described in another paper (Wang et al., PLoS Pathogen, in press). In brief, 293 GnTI- cells were transfected with the dual-expression plasmids (pCAGGS-pur-gQ1/gQ2 and pCAGGS-neo-gHFcHis/gL), and the single clone derived tetramer-expressing cell line was established. The cells were cultivated in a chemically defined protein-free medium, CD293 Medium (Thermofisher Scientific, Waltham, MA) supplemented with 1 *μ*g/ml puromycin and 20 *μ*g/ml gentamicin at 37°C, 5% CO2 for 2 days, and then the culture supernatant was collected, and the tetramer was further purified by Ni-NTA agarose (Qiagen) and size exclusion column chromatography using a Superdex 200 pg column (GE Healthcare, Buckinghamshire, UK).

### 4.2. CpG Adjuvant for Protein Vaccination

CpG containing adjuvant D35/DOTAP was prepared with NanoAssemblr Benchtop (Precision NanoSystems Inc., BC, Canada), which can mediate bottom-up self-assembly for nanoparticle synthesis with microfluidic mixing technology. D35 (an A-type CpG ODN) [33, 34] was dissolved at 500 *μ*g/mL in 25 mM sodium acetate at pH 4.0. One volume of D35 solution and three volumes of 10 mg/mL DOTAP in ethanol were injected into the microfluidic mixer with a combined final flow rate of 15 mL/min (3.75 mL/min ethanol, 11.25 mL/min aqueous). The D35/DOTAP mixtures were immediately dialysed (50 kDa MWCO dialysis tubing; Repligen Corporation, MA) against 5% glucose solution to remove ethanol and unload D35. D35/DOTAP was filtered through a 0.22-*μ*m PVDF filter (Merck KGaA).

For protein immunisation, 10 *μ*g of the gH/gL/gQ1/gQ2 tetrameric complex and 10 *μ*g of D35/DOTAP (containing D35 amount) were mixed and then made up to a volume of 100 *μ*L with phosphate-buffered saline (PBS).

### 4.3. Preparation of PEI-DNA Complex for DNA Vaccination

The construction of expression plasmids for gH, gL, gQ1, and gQ2 (pCAGGS-gH, pCAGGS-gL, pCAGGS-gQ1, and pCAGGS-gQ2) was described previously [11]. The plasmid was amplified in DH5a *Escherichia coli* and purified using QIAGEN EndoFree Plasmid Maxi Kit (QIAGEN), following the manufacturer's instructions. The plasmid DNA was complexed with PEI (87 kDa linear; Polysciences Inc.) based on a method described previously [35]. PEI stock solution was diluted to 1 mg/mL with 5% glucose solution and mixed with an equal volume of glucose solution containing 0.2 mg/mL plasmid DNA to achieve a nitrogen-to-phosphate ratio (N/P) of 7.5. The mixture was incubated for at least 15 min at room temperature for complex formation before use.

### 4.4. Peptide Synthesis

A library containing a total of 162 peptides (No. 1–162) spanning the entire 694-amino-acid (aa) gH sequence, 250-aa gL sequence, 516-aa gQ1 sequence, and 182-aa gQ2 sequence of the HHV-6B HST strain was synthesised by Eurofins Genomics (Tokyo, Japan) as 20-mers overlapping by 10 residues (Supplementary Table 1). All peptides were dissolved in dimethyl sulfoxide (DMSO) at a concentration of 1 mg/ml and stored at −80°C until use. For peptide vaccination, 10 *μ*g of 20-mer peptide and 10 *μ*g of D35/DOTAP were mixed and then made up to a volume of 100 *μ*L with PBS.

### 4.5. Animals and Immunisations

BALB/c and C57BL/6 mice were purchased from CLEA Japan, Inc., and maintained under specific-pathogen-free conditions in accordance with institutional guidelines. Mice of 5–19 weeks of age were used in all of the experiments. All animal experiments were conducted under the approval of the Animal Research Committee of the Research Institute for Microbial Diseases at Osaka University.

Mice were immunised intradermally with 100 *μ*L/dose of the HHV6B tetrameric complex+ D35/DOTAP as protein vaccination or intravenously with 100 *μ*L/dose of PEI-DNA complex as plasmid DNA vaccination. In addition, mice were immunised intradermally with 100 *μ*L/dose of 20-mer peptide + D35/DOTAP as peptide vaccination.

### 4.6. Cell Stimulation with 162 Peptides and IFN-*γ* Assay

One week after the immunisation, spleens were collected and a single-cell suspension was prepared. Red blood cells were lysed at room temperature with 5 mL of ACK lysing buffer for 3 min and washed with RPMI1640. Then, some of the splenocytes were depleted for CD4^+^ or CD8^+^ T cells using the MACS system with mouse CD4 microbeads (L3T4; Miltenyi Biotec) or CD8*α* microbeads (Ly-2; Miltenyi Biotec), in accordance with the manufacturer's instructions. CD4^+^- or CD8^+^-depleted splenocytes and undepleted splenocytes were resuspended in R-10 (RPMI 1640 containing 10% FBS and 5% penicillin and streptomycin) at a concentration of 1 × 10^7^ cells/mL. The splenocytes were seeded at 100 *μ*L/well in 96-well half-area plates (#3696; Corning), in the presence of 10 *μ*g/mL of each of No. 1–162 peptides or 5 *μ*g/mL concanavalin A (Con A) and then cultured at 37°C for 20 h in a 5% CO_2_ incubator. The production of IFN-*γ* in each well was assayed in the supernatants using Mouse IFN-gamma DuoSet ELISA kits (R&D Systems). One experiment for detection of the CD4^+^ or CD8^+^ T-cell response against 162 peptides was assessed using three culture plates. We repeated the experiment at least three times to see the consistency of the responses to each peptide stimulation. For graphical presentation, IFN-*γ* (pg/mL) of each peptide from three culture plates was normalised to the *Z* score (*Z* = x − mean/SD, where *x* is the quantity of IFN-*γ* for each peptide), and the median *Z* score from the three independent experiments was calculated for each peptide. A *Z* score of less than 0.01 was considered to indicate that no response had been detected.

### 4.7. Cell Line

The BW5147 (H2k) lymphoma cell line was transduced retrovirally with a gene encoding one of H2K^d^, H2D^d^, H2L^d^, H2D^b^, and H2K^b^, as described previously [21, 36], and used to determine the CD8^+^ T-cell epitope presenting MHC Ia molecule. The cells were maintained in R-10 in an incubator with a humidified atmosphere containing 5% CO_2_.

### 4.8. Determination of the Restricted MHC Ia Molecule

The CD8^+^ T-cell epitope presenting H2d molecules including H2K^d^, H2D^d^, and H2L^d^ for BALB/c mice, or presenting H2b molecules including H2K^b^ and H2D^b^ for C57BL/6 mice were determined as previously reported [36]. Briefly, BW5147-H2K^d^, -H2D^d^, -H2L^d^, -H2K^b^, or -H2D^b^ cells (4×10^6^ cells) were cocultured with each peptide (10 *μ*g/mL) at 37°C for 1 h. The cells were washed three times with RPMI 1640 medium and resuspended in R-10 at a concentration of 4 × 10^6^ cells/mL. Splenocytes (1 × 10^6^ cells) from immunised mice were stimulated with each peptide-pulsed BW5147 cell line (2 × 10^5^ cells) in 100 *μ*L of R-10 for 20 h at 37°C, and the IFN-*γ* production was determined by ELISA. BW5147-H2K^d^, -H2D^d^, and -H2L^d^ cells were also retrovirally transduced with the gH gene to make BW5147-H2K^d^, -H2D^d^, and -H2L^d^ cells internally expressing gH protein.

## Figures and Tables

**Table 1:** CD8^+^ T-cell short epitope peptide prediction in BALB/c mice.

Peptide no. (glycoprotein)	Length (aa)	Amino acid sequence∗^1^	Estimated scores for restriction molecules∗^2^			%rank		
			BIMAS			NetMHC		
			D^d^	K^d^	L^d^	D^d^	K^d^	L^d^
No. 13 (gH)	20	IVYSLNLYPSHGIYYIRVVE						
	10	**LYPSHGIYYI**	**10**	**2880**	1.95	0.8	**4**	1.9
	9	LNLYPSHGI	0.5	80	1	27	10	37
	9	VYSLNLYPS	0.1	60	1	60	9.5	20
	9	LYPSHGIYY	2	60	3	**0.7**	17	13
	8	LYPSHGIY	2	60	—	3	18	35
	9	YPSHGIYYI	0.5	57.6	**39**	3.5	13	**0.17**
	8	NLYPSHGI	0.5	57.6	—	36	17	32
	10	SLNLYPSHGI	0.6	40	1	55	18	55
	9	HGIYYIRVV	6	20	2	7.5	33	50
	8	HGIYYIRV	6	20	—	12	55	37

No. 113 (gQ1)^∗^^3^	20	RLKPLTAMTAIAFCPMTSKL						
	9	**AFCPMTSKL**	1	1382.4	5	37	0.8	23

No. 157 (gQ2)	20	NGPRYLQMETFISDLFRYEC						
	9	**RYLQMETFI**	0.3	**5760**	1	11	**0.01**	5
	8	RYLQMETF	0.18	144	—	9.5	0.8	16
	10	RYLQMETFIS	0.12	100	1	28	0.12	31
	10	LQMETFISDL	1.2	96	5	55	16	**2.5**
	9	TFISDLFRY	0.1	57.6	2	45	26	37
	9	QMETFISDL	1	48	1.5	60	12	4.5
	8	YLQMETFI	1.5	40	—	29	0.05	12
	10	TFISDLFRYE	0.01	6.912	0.1	55	33	75
	8	TFISDLFR	0.01	5.76	—	80	48	80
	8	NGPRYLQM	**120**	5	—	**0.08**	75	37
	10	GPRYLQMETF	0.36	1	**90**	1	75	8.5

∗^1^ Underlined peptides were synthesised and used for experiments. Bold type peptides indicate determined short CD8^+^ T-cell epitope. ∗^2^ No binding score. ∗^3^ AFCPMTSKL in No.113 was previously reported as the peptide including CD8^+^ T-cell epitope (Nagamata et al., 2019).

**Table 2:** CD8^+^ T-cell short epitope peptide prediction in C57BL/6 mice.

Peptide no. (glycoprotein)	Length (aa)	Amino acid sequence∗1	Estimated scores for restriction molecules^∗^^2^		%rank	
			BIMAS		NetMHC	
			D^b^	K^b^	D^b^	K^b^
No. 133 (gQ1)	20	QRGTSIRNIDPAIVSALWHS				
	9	IDPAIVSAL	0.017	**3.3**	26	18
	8	DPAIVSAL	—	1.1	65	70
	8	RGTSIRNI	—	0.475	48	30
	10	NIDPAIVSAL	0.05	0.24	27	46
	8	RNIDPAIV	—	0.174	48	19
	10	RNIDPAIVSA	0.119	0.158	55	50
	9	AIVSALWHS	0.022	0.132	35	40
	9	**TSIRNIDPA**	**1047.388**	0.12	**0.01**	**11**

No. 141 (gQ1)	20	MFEKKIYGYEHLGQALCEGG				
	8	KIYGYEHL	—	**132**	9	**0.03**
	10	YGYEHLGQAL	**3.024**	8.64	**1.2**	1.8
	9	KKIYGYEHL	0.899	6	4.5	5
	10	EKKIYGYEHL	0.011	1.2	13	22
	8	YEHLGQAL	—	1	21	43
	9	YGYEHLGQA	0.108	0.72	7.5	3.5
	9	GYEHLGQAL	0.006	0.24	35	34
	8	EHLGQALC	—	0.11	95	80

No. 157 (gQ2)	20	NGPRYLQMETFISDLFRYEC				
	8	NGPRYLQM	—	**103.68**	23	4.5
	10	LQMETFISDL	**15.459**	1.32	**3.5**	6
	8	METFISDL	—	1.1	37	28
	10	NGPRYLQMET	0.108	0.432	36	60
	10	YLQMETFISD	0.059	0.3	28	43
	8	LQMETFIS	—	0.264	39	37
	9	QMETFISDL	1.175	0.24	16	**2.5**
	10	RYLQMETFIS	0.003	0.22	4	36

∗^1^ Underlined peptides were synthesised and used for experiments. Bold type peptides indicate determined short CD8^+^ T-cell epitope. ∗^2^ No binding score.

## Data Availability

All data generated or analysed during this study are included in this published article and its Supplementary Information files.

## Supplementary Materials

Supplementary Materials Supplementary Table 1: peptide library used in this study. Supplementary Figure 1: (a) C57BL/6 mice were immunised at the tail base with 40 *μ*g of influenza HA split vaccine with or without D35/DOTAP (D35: 10 *μ*g) adjuvant. Seven days after immunisation, splenocytes were stimulated with 5 *μ*g/mL A/California/7/2009 (X-179A) (H1N1) pdm09 or 5 *μ*g/mL epitope peptide ASNENMETM, which is the H2-Db restricted epitope peptide within the NP of influenza A/PR/8/34 (H1N1). After 20 h of stimulation, the production of IFN-*γ* was measured by ELISA, and data are presented in pg/mL. Note that influenza HA split vaccine is a mixture of equal amounts of A/California/7/2009 (X179A) (H1N1) pdm09, A/Hong Kong/4801/2014 (X-263) (H3N2), B/Phuket/3073/2013 (Yamagata), and B/Texas/2/2013 (Victoria) provided from the Research Foundation for Microbial Diseases of Osaka University. (b) BALB/c mice were immunised at the tail base with 10 *μ*g of LLO91-99 peptide (GYKDGNEYI) with or without D35/DOTAP (D35: 10 *μ*g) adjuvant. Seven days after immunisation, splenocytes were stimulated with 5 *μ*g/mL LLO91-99 peptide. After 20 h of stimulation, the production of IFN-*γ* was measured by ELISA, and data are presented in pg/mL. Supplementary Figure 2: short peptide stimulation and the restricted MHC I molecule determination after expression plasmid DNA vaccination in C57BL/6 mice. (a) C57BL/6 splenocytes immunised with a mixture of gH, gL, gQ1, and gQ2 expression plasmids were stimulated with the indicated CD8+ T-cell short epitope candidate peptides. After 20 h of stimulation, IFN-*γ* (pg/mL) of each peptide was measured by ELISA (A; left). The restricted MHC I molecules were examined by each peptide-pulsed BW5147 cell line expressing H2Db or H2Kb (a; right). (b) C57BL/6 splenocytes immunised with a mixture of gH, gL, gQ1, and gQ2 expression plasmids were first depleted of CD4+ T cells (to increase the detection sensitivity of CD8+ T-cell responses) and then stimulated with the indicated CD8+ T-cell short epitope candidate peptides. After 20 h of stimulation, IFN-*γ* (pg/mL) of each peptide was measured by ELISA (b; left). The restricted MHC I molecules were examined with each peptide-pulsed BW5147 cell line expressing H2Db or H2Kb (b; right).
