# Supplementary material for: Comprehensive Screening of Mouse T-Cell Epitopes in Human Herpesvirus 6B Glycoprotein H/L/Q1/Q2 Tetramer Complex
Source: J Immunol Res. 2020 Jul 26;2020:4697529. doi: 10.1155/2020/4697529 (PMC7399772; doi:10.1155/2020/4697529)
Supplement: Supplementary Materials — Supplementary Table 1: peptide library used in this study. Supplementary Figure 1: (a) C57BL/6 mice were immunised at the tail base with 40 μg of influenza HA split vaccine with or without D35/DOTAP (D35: 10 μg) adjuvant. Seven days after immunisation, splenocytes were stimulated with 5 μg/mL A/California/7/2009 (X-179A) (H1N1) pdm09 or 5 μg/mL epitope peptide ASNENMETM, which is the H2-Db restricted epitope peptide within the NP of influenza A/PR/8/34 (H1N1). After 20 h of stimulation, the production of IFN-γ was measured by ELISA, and data are presented in pg/mL. Note that influenza HA split vaccine is a mixture of equal amounts of A/California/7/2009 (X179A) (H1N1) pdm09, A/Hong Kong/4801/2014 (X-263) (H3N2), B/Phuket/3073/2013 (Yamagata), and B/Texas/2/2013 (Victoria) provided from the Research Foundation for Microbial Diseases of Osaka University. (b) BALB/c mice were immunised at the tail base with 10 μg of LLO91-99 peptide (GYKDGNEYI) with or without D35/DOTAP (D35: 10 μg) adjuvant. Seven days after immunisation, splenocytes were stimulated with 5 μg/mL LLO91-99 peptide. After 20 h of stimulation, the production of IFN-γ was measured by ELISA, and data are presented in pg/mL. Supplementary Figure 2: short peptide stimulation and the restricted MHC I molecule determination after expression plasmid DNA vaccination in C57BL/6 mice. (a) C57BL/6 splenocytes immunised with a mixture of gH, gL, gQ1, and gQ2 expression plasmids were stimulated with the indicated CD8+ T-cell short epitope candidate peptides. After 20 h of stimulation, IFN-γ (pg/mL) of each peptide was measured by ELISA (A; left). The restricted MHC I molecules were examined by each peptide-pulsed BW5147 cell line expressing H2Db or H2Kb (a; right). (b) C57BL/6 splenocytes immunised with a mixture of gH, gL, gQ1, and gQ2 expression plasmids were first depleted of CD4+ T cells (to increase the detection sensitivity of CD8+ T-cell responses) and then stimulated with the indicated CD8+ T-cell short epitop [file 4697529.f1.pdf]

Supplementary Table 1. Peptide library used in this study

	No.	Peptide sequence		No.	Peptide sequence
gH	1	MLFRLWVFVLLTPCYSWRPW		81	PESRDNWLNFFNGFQTCSPS
	2	LTPCYSWRPWTISNESHCKN		82	FNGFQTCSPSVGITTCISDN
	3	TISNESHCKNGNSENPIVRP		83	VGITTCISDNCRKYLPERIT
	4	GNSENPIVRPGFITFNFYTK		84	CRKYLPERITYVNNFFVDNI
	5	GFITFNFYTKNDTRIYQVPK		85	YVNNFFVDNIAGLEFNISEN
	6	NDTRIYQVPKCLLGSDITYH		86	AGLEFNISENTDSFYSNIGF
	7	CLLGSDITYHLFDAINTTES		87	TDSFYSNIGFLLYLENPATG
	8	LFDAINTTESLTNYEKRVTR		88	LLYLENPATGITKIIRFPFN
	9	LTNYEKRVTRFYEPPMNDIL		89	ITKIIRFPFNSLTLFDTILN
	10	FYEPPMNDILRLSTVPAVKQ		90	SLTLFDTILNCLKYFHLKTG
	11	RLSTVPAVKQFNLDHSIQPQ		91	CLKYFHLKTGVEFDLLKQME
	12	FNLDHSIQPQIVYSLNLYPS		92	VEFDLLKQMEAYNSKLPFRS
	13	IVYSLNLYPSHGIYYIRVVE		93	AYNSKLPFRSSRPTILIRNT
	14	HGIYYIRVVEVRQMQYDNVS	gQ1	94	MRPPRCSAPILVCAISMATA
	15	VRQMQYDNVSCKLPNSLNEL		95	LVCAISMATALSNATVYRDA
	16	CKLPNSLNELIFPVQVRCAK		96	LSNATVYRDAGTVESTPPPD
	17	IFPVQVRCAKITRYAGENIY		97	GTVESTPPPDDEDNYTAKYY
	18	ITRYAGENIYTHFFTPDFMI		98	DEDNYTAKYYDDSIYFNIYD
	19	THFFTPDFMILYIQNPAGDL		99	DDSIYFNIYDGTNPTPRRRT
	20	LYIQNPAGDLTMMYGNTTDI		100	GTNPTPRRRTLPEIISKFST
	21	TMMYGNTTDINFKAPYRKSS		101	LPEIISKFSTSEMSRLGGLK
	22	NFKAPYRKSSFIFKQTLTDD		102	SEMSRLGGLKVFVPVDYTPT
	23	FIFKQTLTDDLLLIVEKDVV		103	VFVPVDYTPTTTLEDIEDLL
	24	LLLIVEKDVVDEEYRFISDA		104	TTLEDIEDLLNYAICDDNSC
	25	DEEYRFISDATFVDETLDDV		105	NYAICDDNSCGCLIETEARI
	26	TFVDETLDDVDEVEALLLKF		106	GCLIETEARIMFGDIIICVP
	27	DEVEALLLKFNNLGIQTLLR		107	MFGDIIICVPLSAESRGVRN
	28	NNLGIQTLLRGDCKKPDYAG		108	LSAESRGVRNLKNRLMPMGL
	29	GDCKKPDYAGIPQMMFLYGI		109	LKNRLMPMGLSQILSSGLGL
	30	IPQMMFLYGIVHFSYSTKNT		110	SQILSSGLGLHFSLLYGAFG
	31	VHFSYSTKNTGPMPVLRVLK		111	HFSLLYGAFGSNYNSLAYME
	32	GPMPVLRVLKTHENLLSIDS		112	SNYNSLAYMERLKPLTAMTA
	33	THENLLSIDSFVNRCVNVSE		113	RLKPLTAMTAIAFCPMTSKL
	34	FVNRCVNVSEGTIQYPKMKE		114	IAFCPMTSKLELRQNYRLEK
	35	GTIQYPKMKEFLKYEPSDYS		115	ELRQNYRLEKARCELIVNIE
	36	FLKYEPSDYSYITKNKSIPV		116	ARCELIVNIELLKIQNHGGQ
	37	YITKNKSIPVSTLLTYLATA		117	LLKIQNHGGQTIKTLTSFAI
	38	STLLTYLATAYETNVTISRY		118	TIKTLTSFAIVRKDNDGQDW
	39	YETNVTISRYKWSDIANTLQ		119	VRKDNDGQDWETCTRFASVS
	40	KWSDIANTLQKIYEKHMFFT		120	ETCTRFASVSIEDILKSKPA
	41	KIYEKHMFFTNLTFSDRETL		121	IEDILKSKPAANGTCCPPRD
	42	NLTFSDRETLFMLAEIANFI		122	ANGTCCPPRDVHHDRPTLQS
	43	FMLAEIANFIPADERMQRHM		123	VHHDRPTLQSSNSWTRTEYF
	44	PADERMQRHMQLLIGNLCNP		124	SNSWTRTEYFEPWQDVVDAY
	45	QLLIGNLCNPVEIVSWAHML		125	EPWQDVVDAYVPINDNHCPN
	46	VEIVSWAHMLTADKAPNLEN		126	VPINDNHCPNDSYVVFQTLQ
	47	TADKAPNLENIYSPCASPVR		127	DSYVVFQTLQGHEWCSRLNK
	48	IYSPCASPVRRDVTNSFVKT		128	GHEWCSRLNKNDTKNYLSSV
	49	RDVTNSFVKTVLTYASLDRY		129	NDTKNYLSSVLAFKNALYET
	50	VLTYASLDRYRSDMMEMLSV		130	LAFKNALYETEELMETIGMR
	51	RSDMMEMLSVYRPPDMARVA		131	EELMETIGMRLASQILSLVG
	52	YRPPDMARVAAIQCLSPSEP		132	LASQILSLVGQRGTSIRNID
	53	AIQCLSPSEPAASLPLPNVT		133	QRGTSIRNIDPAIVSALWHS
	54	AASLPLPNVTFVISPSYVIK		134	PAIVSALWHSLPEKLTTTNI
	55	FVISPSYVIKGVSLTITTTI		135	LPEKLTTTNIKYDIASPTHM
	56	GVSLTITTTIVATSIIITAI		136	KYDIASPTHMSPALCTIFVQ
	57	VATSIIITAIPLNSTCVSTN		137	SPALCTIFVQTGTSKQRFRN
	58	PLNSTCVSTNYKYAGQDLLV		138	TGTSKQRFRNAGLLMVNNIF
	59	YKYAGQDLLVLRNISSQTCE		139	AGLLMVNNIFTVQARYSKQN
	60	LRNISSQTCEFCQSVVMEYD		140	TVQARYSKQNMFEKKIYGYE
	61	FCQSVVMEYDDIDGPLQYIY		141	MFEKKIYGYEHLGQALCEGG
	62	DIDGPLQYIYIKNIDELKTL		142	HLGQALCEGGHVFYNPRDVY
	63	IKNIDELKTLTDPNNNLLVP		143	HVFYNPRDVYFQNIKMAATE
	64	TDPNNNLLVPNTRTHYLLLA		144	FQNIKMAATEPTVVRT
	65	NTRTHYLLLAKNGSVFEMSE	gQ2	145	MHFVAVYILTHFHAYPGVAA
	66	KNGSVFEMSEVGIDIDQVSI		146	HFHAYPGVAALPFFSTLPKI
	67	VGIDIDQVSIILVIIYVLIA		147	LPFFSTLPKITSCCDHYVVL
	68	ILVIIYVLIAIIALFGLYRL		148	TSCCDHYVVLNSLSSVSSST
	69	IIALFGLYRLIRLC		149	NSLSSVSSSTPTCLDGEILF
gL	70	MELLLFVMSLILLTFSKAMP		150	PTCLDGEILFQNAGQKFCRP
	71	ILLTFSKAMPLFDHNSFYFE		151	QNAGQKFCRPFTDNRTIVYT
	72	LFDHNSFYFEKLDDCIAAVI		152	FTDNRTIVYTMQDQVQRPWS
	73	KLDDCIAAVINCTRSEVPLL		153	MQDQVQRPWSVTWMDFNLVI
	74	NCTRSEVPLLLEPIYQAPVY		154	VTWMDFNLVISDYGRAVIEN
	75	LEPIYQAPVYNEDVMSILLK		155	SDYGRAVIENLTESAMSAHK
	76	NEDVMSILLKPPTKKKPFSR		156	LTESAMSAHKNGPRYLQMET
	77	PPTKKKPFSRIMVTNEFLSD		157	NGPRYLQMETFISDLFRYEC
	78	IMVTNEFLSDFLLLQDNPEQ		158	FISDLFRYECHRDNRYVLEK
	79	FLLLQDNPEQLRTLFALIGD		159	HRDNRYVLEKKLQMFYPTTH
	80	LRTLFALIGDPESRDNWLNF		160	KLQMFYPTTHMNELLFYPSD
				161	MNELLFYPSDPTLPSPYGNG
				162	PTLPSPYGNGHY

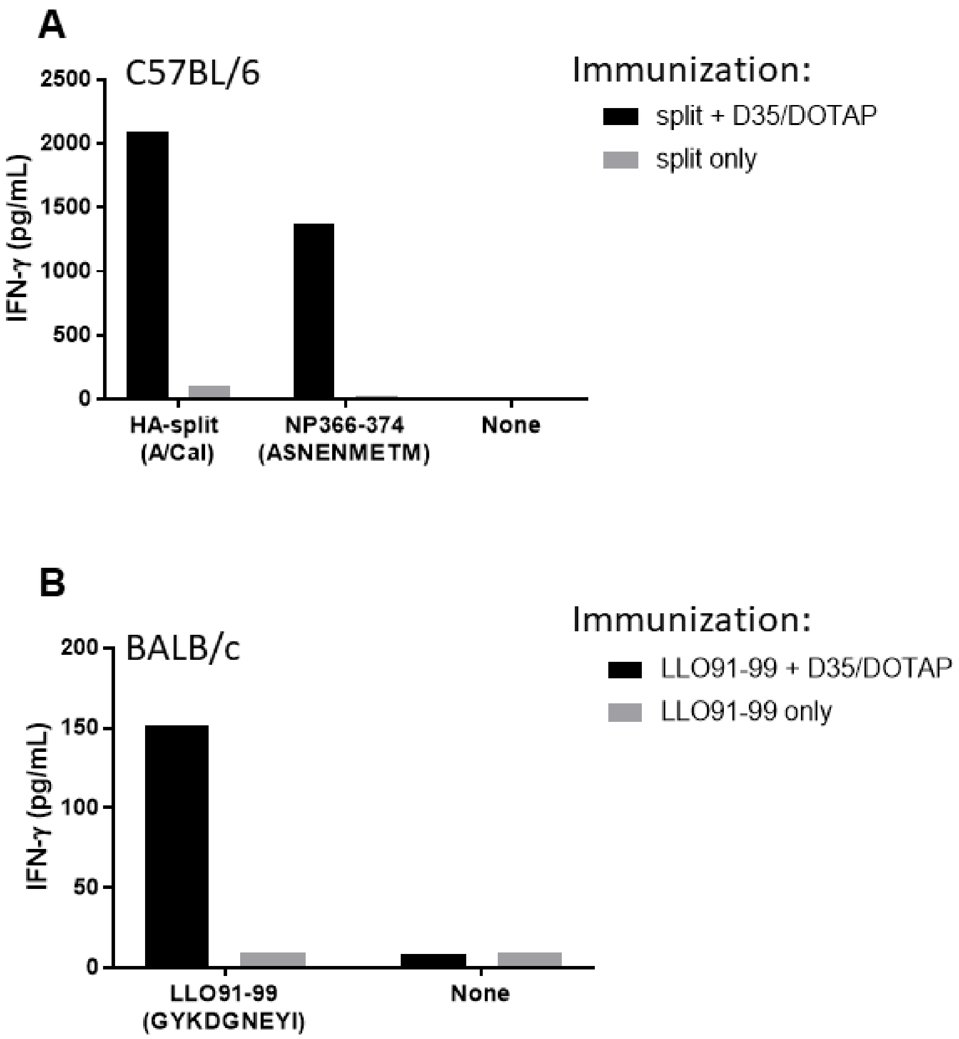

Supplementary Figure 1.

(A) C57BL/6 mice were immunised at the tail base with 40 μg of influenza HA split vaccine with or without D35/DOTAP (D35: 10 μg) adjuvant. Seven days after immunisation, splenocytes were stimulated with 5 μg/mL A/California/7/2009 (X-179A) (H1N1) pdm09 or 5 μg/mL epitope peptide ASNENMETM, which is the H2-Db restricted epitope peptide within the NP of Influenza A/PR/8/34 (H1N1). After 20 h of stimulation, the production of IFN-γ was measured by ELISA and data are presented in pg/mL.

Note that influenza HA split vaccine is a mixture of equal amounts of A/California/7/2009 (X-179A) (H1N1) pdm09, A/Hong Kong/4801/2014 (X-263) (H3N2), B/Phuket/3073/2013 (Yamagata), and B/Texas/2/2013 (Victoria) provided from the Research Foundation for Microbial Diseases of Osaka University.

(B) BALB/c mice were immunised at the tail base with 10 μg of LLO91-99 peptide (GYKDGNEYI) with or without D35/DOTAP (D35: 10 μg) adjuvant. Seven days after immunisation, splenocytes were stimulated with 5 μg/mL LLO91-99 peptide. After 20 h of stimulation, the production of IFN-γ was measured by ELISA and data are presented in pg/mL.

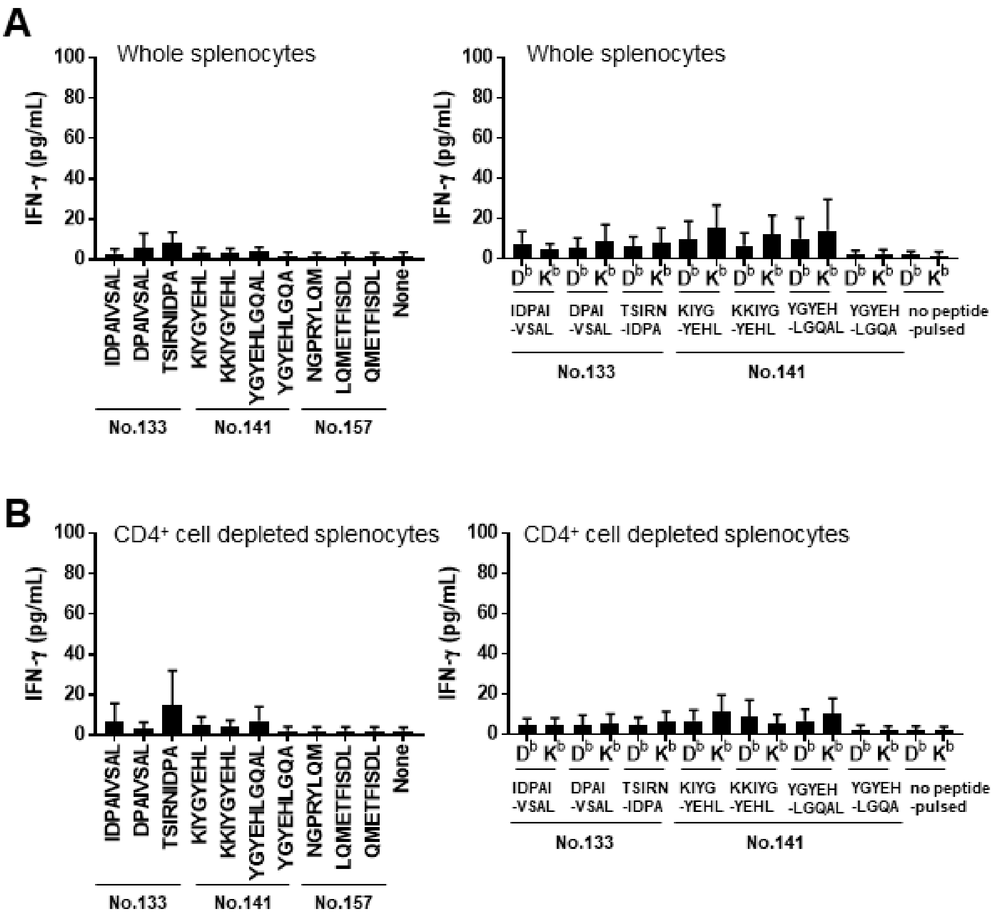

Supplementary Figure 2. Short peptide stimulation and the restricted MHC I molecule determination after expression plasmid DNA vaccination in C57BL/6 mice

(A) C57BL/6 splenocytes immunised with a mixture of gH, gL, gQ1, and gQ2 expression plasmids were stimulated with the indicated $CD8^+$ T-cell short epitope candidate peptides. After 20 h of stimulation, IFN-γ (pg/mL) of each peptide was measured by ELISA (A; left). The restricted MHC I molecules were examined by each peptide-pulsed BW5147 cell line expressing H2Db or H2Kb (A; right).

(B) C57BL/6 splenocytes immunised with a mixture of gH, gL, gQ1, and gQ2 expression plasmids were first depleted of $CD4^+$ T cells (to increase the detection sensitivity of $CD8^+$ T-cell responses), and then stimulated with the indicated $CD8^+$ T-cell short epitope candidate peptides. After 20 h of stimulation, IFN-γ (pg/mL) of each peptide was measured by ELISA (B; left). The restricted MHC I molecules were examined with each peptide-pulsed BW5147 cell line expressing H2Db or H2Kb (B; right).
